# miRMaster 2.0: multi-species non-coding RNA sequencing analyses at scale

**DOI:** 10.1093/nar/gkab268

**Published:** 2021-04-19

**Authors:** Tobias Fehlmann, Fabian Kern, Omar Laham, Christina Backes, Jeffrey Solomon, Pascal Hirsch, Carsten Volz, Rolf Müller, Andreas Keller

**Affiliations:** Chair for Clinical Bioinformatics, Saarland University, 66123 Saarbrücken, Germany; Chair for Clinical Bioinformatics, Saarland University, 66123 Saarbrücken, Germany; Chair for Clinical Bioinformatics, Saarland University, 66123 Saarbrücken, Germany; Chair for Clinical Bioinformatics, Saarland University, 66123 Saarbrücken, Germany; Chair for Clinical Bioinformatics, Saarland University, 66123 Saarbrücken, Germany; Chair for Clinical Bioinformatics, Saarland University, 66123 Saarbrücken, Germany; Department of Microbial Natural Products, Helmholtz-Institute for Pharmaceutical Research Saarland (HIPS), Helmholtz Centre for Infection Research (HZI) and Department of Pharmacy, Saarland University, Campus E8 1, 66123 Saarbrücken, Germany; Department of Microbial Natural Products, Helmholtz-Institute for Pharmaceutical Research Saarland (HIPS), Helmholtz Centre for Infection Research (HZI) and Department of Pharmacy, Saarland University, Campus E8 1, 66123 Saarbrücken, Germany; Chair for Clinical Bioinformatics, Saarland University, 66123 Saarbrücken, Germany; Department of Neurology and Neurological Sciences, Stanford University School of Medicine, Stanford, CA, USA

## Abstract

Analyzing all features of small non-coding RNA sequencing data can be demanding and challenging. To facilitate this process, we developed miRMaster. After the analysis of over 125 000 human samples and 1.5 trillion human small RNA reads over 4 years, we present miRMaster 2 with a wide range of updates and new features. We extended our reference data sets so that miRMaster 2 now supports the analysis of eight species (e.g. human, mouse, chicken, dog, cow) and 10 non-coding RNA classes (e.g. microRNAs, piRNAs, tRNAs, rRNAs, circRNAs). We also incorporated new downstream analysis modules such as batch effect analysis or sample embeddings using UMAP, and updated annotation data bases included by default (miRBase, Ensembl, GtRNAdb). To accommodate the increasing popularity of single cell small-RNA sequencing data, we incorporated a module for unique molecular identifier (UMI) processing. Further, the output tables and graphics have been improved based on user feedback and new output formats that emerged in the community are now supported (e.g. miRGFF3). Finally, we integrated differential expression analysis with the miRNA enrichment analysis tool miEAA. miRMaster is freely available at https://www.ccb.uni-saarland.de/mirmaster2.

## Graphical Abstract

**Graphical Abstract:**
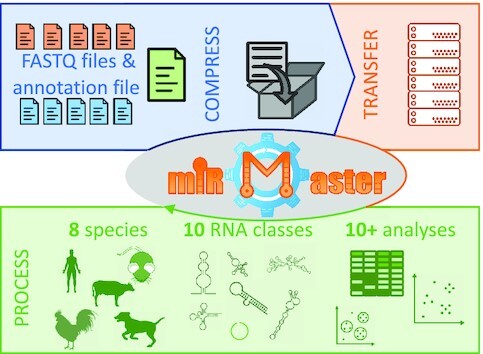
miRMaster 2 workflow. The workflow is split into three steps: data selection and compression, data upload and server-side processing.

## INTRODUCTION

The reliable analysis of small non-coding RNA (sncRNAs) sequencing data can be challenging, time consuming and varies in many aspects. This includes the primary processing of sequencing data for quality control but also downstream analyses. Besides tRNAs and snoRNAs, microRNAs are sncRNAs that are extensively studied already for over two decades. A comprehensive summary on the state-of-the art in microRNA biology has been published by Bartel in 2018 (1). In addition to the canonical biology of microRNAs, more and more non-canonical aspects of miRNA biology are becoming obvious (2), calling for a broad variety of analysis aspects. It is thus not surprising, that many miRNA analysis tools, online and stand-alone, are available. The current release of Aviator (https://www.ccb.uni-saarland.de/aviator), a tool that aims to provide accessibility statistics of all web servers and data bases in life sciences, lists 322 web-based resources for microRNAs, of which 235 are currently working. By developing miRMaster (3,4), we provide a tool with a strong focus on the analysis of all aspects of miRNAs described in a systematic manner. While the tool became stepwisely broader in its functionality aspect to cover other sncRNA classes, microRNAs are still its anchor point. Based on the original version of miRMaster, 1500 runs have been completed, over 125 000 human samples have been analyzed, and 1.5 trillion human small RNA reads were processed over four years. To make further use of the uploaded data, we ask miRMaster users whether we can re-analyze the aggregated sequencing reads as further feedback for the development of our tool and as comparison to our small RNA research projects.

As mentioned before, several other web servers and web-services for analyzing miRNA sequencing data exist, overlapping partially with miRMaster's functionality. We thus want to put our tool in the context of others and recent developments in the field. A broad tool meta review has been published by Chen *et al.*, which covers 95 review papers and about 1000 miRNA bioinformatics tools (5). The variety of tools reaches from rather specialized tools, e.g. for the detection of isomiRs or miRNA editing (6) to very broad analysis pipelines. Among the tools with broader analysis functionality with a particular focus on miRNAs, we want to mention CBS-miRSeq (7), miRquant (8), sRNAbench/sRNAtoolbox (9), Chimira (10), mirPRo (11), miRge (12) and CPSS2 (13). Among those, sRNAtoolbox was updated most recently with improvements made to e.g. the batch processing, library preparation protocols and differential expression analysis. Moreover, SPAR is a small RNA-seq portal for analysis of sequencing experiments (14). GLASSgo for example facilitates automated detection of sRNA homologs (15). Also, for viruses, sRNA analysis tools have been developed such as MISIS-2, a tool for the in-depth analysis of sRNAs and representation of consensus master genomes in viral quasispecies (16). With respect to non-canonical miRNA biology, such as miRNA sponges (17), also specialized software tools have been made available (18). Another aspect is the analysis of miRNA editing and chemical modifications. Likewise, for this task, specialized tools are available such as Prost! (19), DeAnniso (20) and others. Similarly, for tRNAs and other ncRNA classes, chemical modifications exist (21) that further complicate the analysis of ncRNA data sets.

The number of available tools, web-based and standalone, reflects the continuous interest of the research community in non-coding RNAs. At the same time, it pinpoints that a more integrative analysis of different ncRNA classes is required. Recently, a ‘changing of the guards’ model has been proposed, where microRNA levels decreased but small transfer RNA fragments increased in blood of patients (22).

**Figure 1.**
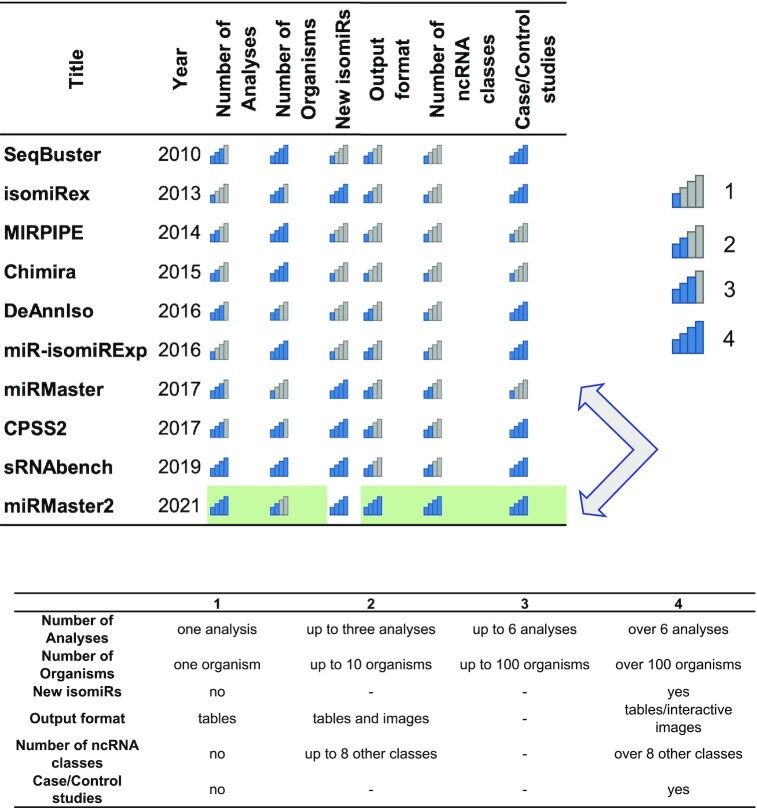
Tool comparison. Comparison of features provided by tools analyzing sncRNA-seq data. Improvements of miRMaster 2 in comparison to its original release are marked in green.

Although a direct comparison in terms of scope, functionality, ease of use and other parameters is challenging and partially subjective, we aimed to provide at least an overview on a selection of commonly used broader analysis tools that are available as web-service. We thus evaluated the number of analyses, the number of supported organisms, the number of supported ncRNA classes and other parameters for 10 tools and present the result sorted by the publication date (Figure 1). The analysis reveals an expected pattern, the more recent tools have a broader scope of functionality as compared to the early tools. The recently updated sRNABench for example excels in basically all categories, e.g. offers more organisms as compared to miRMaster 2. miRMaster 2 offers in contrast more output options and covers more ncRNA classes. Figure 1 allows to compare the functionality of the 10 selected tools and supports users in their decision to select one tool.

## MATERIALS AND METHODS

### Reference data bases

miRMaster relies on public annotation data sets of several well-known and widely used reference data bases. These include for the different RNAs miRBase (version 22.1 (23)), Ensembl ncRNA (version 100 (24)), RNACentral (for piRNAs) (version 15 (25)), GtRNAdb (version 18.1 (26)), circBase (accessed 25.10.20 (27)), NONCODE (version 5 (28)) and NCBI RefSeq for the reference genomes as well as viruses and bacteria.

### Supported sequencing protocols

miRMaster 2 directly supports the most common sequencing protocols. This includes Illumina Truseq, Bioo Scientific Nextflex, MGISeq and Diagenodes D-Plex and CATS technology.

### Implementation and graphical representation of the web service

The miRMaster 2 web service was implemented using Python 3.7.6 with Django 2.2.10, Postgres 11.1 and Redis 5.0. All services are encapsulated in docker containers and bundled with docker compose. The job queue is handled with Celery 4.4.7 and Redis, and the jobs are executed via Snakemake 5.31.1 (29). The frontend was styled with Bootstrap 4.5.3 and the interactive features are based on the Angular JS 1.5.11 and jQuery 3.4.1 libraries. Plots are rendered with Highcharts 8.2.2 and Clustergrammer-GL 0.22.0 (30). RNA secondary structures are rendered with fornac 1.1.10 and interactive tables with DataTables 1.10.23.

### Compression and quality control data

miRMaster accepts raw FASTQ and gzip compressed FASTQ files as input. At first and before the data is sent to our server, we perform three pre-processing steps encompassing adapter trimming, quality filtering and read collapsing via JavaScript on the upload page. The collapsed reads are transmitted to the servers as soon as possible but in chunks of ∼16MB, thereby ensuring a low RAM consumption. The quality scores of the reads are collected on the user side and only aggregated metrics are sent to the server. Multiple annotations can be provided for the uploaded samples, which can then be highlighted in the resulting reports. In particular, a group annotation for differential expression downstream can be selected, in which case miRMaster will perform additional analyses.

### Data pre-processing

While most of the analysis parameters in the pre-processing view are conveniently set to reasonable default values, the expert mode allows full control and maximal flexibility. In the pre-processing view, the 5′ barcode length, an adapter barcode length and the length of the unique molecular identifier (UMI) can be determined. Further, after activation of the expert mode, minimum read length and maximum adapter edit distance can be modified as well as the minimum read/adapter overlap. Finally, leading and trailing N’s can be trimmed, or reads containing N’s can be fully omitted. For quality trimming, the sliding window size and quality can be selected. To maximize the processing capabilities of the user's CPU, the number of threads that can be used on the client side for the pre-processing can be configured.

### Mapping

The read mapping process in miRMaster 2 is carried out using Bowtie 1.2.3 (31) as the standard option. For the update we added STAR 2.7.5a (32) as alternative mapper. In the standard mode, up to five hits in the reference genome are allowed for each read, where the mapping seed length is set to 18 nt and no mismatches in the seed region are allowed. In the expert mode, the user is free to change these parameters. For quantifying miRNAs, reads are mapped against miRNA precursors while allowing per default one mismatch. The resulting mappings are then filtered to count only reads mapping to the annotated miRNAs with at most two nucleotides differences at the 5′ end and five nucleotides at the 3′ end. For isomiR quantification, the mapping is performed with one additional mismatch and then subsequently filtered, such that non-templated nucleotide additions are not counted as mismatches. Other ncRNAs are per default quantified without any mismatches and all multi-mapping reads with the lowest number of mismatches are considered. To generate the miRGFF3 isoform format, mirtop 0.4.23 (33) is run on the aligned files and subsequently filtered to accommodate the user selected number of allowed mismatches.

### Detection filtering

Before applying analysis methods to the expression matrices of the different ncRNA types, these matrices are filtered per default, such that only those RNAs that are expressed in at least 50% of all the samples are kept, or in case a differential expression annotation variable is provided (e.g. Diagnosis), in at least 50% of the samples of one of the variable levels (e.g. Dementia or Control). For this filtering procedure, only RNAs that are expressed with at least three reads are considered detected. In addition to normalizing the reads by the sequencing depth (RPM) we log_2_ transform the expression data and add a pseudo count of one.

### Embedding

miRMaster 2 is equipped with two common dimension reduction approaches, namely Principal Component Analysis (PCA) and Uniform Manifold Approximation and Projection (UMAP). The user can first select the embedding from a drop down followed by the response variable. This response variable is extracted from the annotation file and the data points are colored according to the respective grouping. As representation, 2D-scatter plots are provided to the user.

### Clustering

Hierarchical clustering with Euclidean distance and complete linkage is performed on the sample Spearman correlation, which is determined based on the reads per million (RPM) normalized expression matrix for each ncRNA type separately, as well as for all sncRNAs. In addition, hierarchical clustering is also performed on the RPM log_2_ normalized expression matrix for each ncRNA type, as well as on subsets of the top RNAs with the largest variance.

### Batch effect analysis

To detect the influence of technical batches or attribute variance to biologically relevant parameters, a Principal Variance Component Analysis (PVCA) is performed. PVCA combines the strengths of two data analysis techniques, principal component analysis (PCA), which reduces the feature dimensions while maintaining the largest fraction of the variability in the data, and variance components analysis (VCA), which fits a mixed linear model using factors of interest as random effects. In more detail, all variables provided in the annotation file are fit as random effects including two-way interaction terms in the mixed model. Thereby, principal components obtained from the original data expression matrix are selected. As a result, the proportion of variance that is attributed to the variables from the annotation file is reported.

### Differential gene expression analysis

The groupings for differential expression analysis are extracted from the annotation file provided by the user. If more than two groups are given, all pair-wise differential expression analyses are performed automatically. miRMaster first computes whether the features are normally distributed by applying the Shapiro Wilk test. As hypothesis test for assessing the degree of differential expression, *t*-test (for normally distributed data) and Wilcoxon Mann–Whitney test otherwise, are calculated. For multiple groups, also analysis of variance (ANOVA) as well as the non-parametric Kruskal–Wallis test are computed. The *P*-values are adjusted for multiple testing by controlling the false discovery rate using the Benjamini–Hochberg procedure. Further measures representing effect sizes are fold changes, the area under the receiver operator characteristics curve (AUC value) and Cohen's *d*. In addition to tabular output, volcano plots (log_2_ fold change versus negative decade logarithm of *P*-values) are displayed and boxplots per RNA are shown.

### miRNA prediction

The prediction of new miRNAs follows the same principles as described in miRDeep (34) and our previous publications (3,4). Similarly, the expert mode allows maximal flexibility. As a first step, after the reads have been mapped to the genome, miRNA precursor candidates are determined. To this end local maximum read stacks, which are assumed to stem from potential miRNAs, are searched in downstream windows of per default 70 nucleotides and two precursors are excised from each stack. For this step, the required minimum read stack can be increased in order to improve the specificity of miRNA precursor predictions. Also, minimal and maximal length of the mature miRNA(s) can be set. Moreover, the mapping fraction consistent with Dicer processing can be increased or decreased. miRMaster groups new miRNA candidates into several categories, depending on their overlap with known miRNAs or known miRNA precursors. Precursor miRNA candidates belonging to the ‘novel’ category are not overlapping any known miRNAs and none of their annotated miRNAs have a similarity to any known miRNA of the same species. Details on the different categories are provided in the software tutorial page. To further rank and prioritize the predicted precursors, NovoMiRank (35) is subsequently applied and the scores are presented in the results table.

### API to miEAA

Following our ambition to develop a fully integrated knowledge base on miRNAs (36), we started to integrate our tools such as miEAA (37,38) and miRSwitch (39) with APIs. For miRMaster we continued this process in the reverse direction. miRNAs can be sorted by their expression level, or if case–control studies are evaluated, also with respect to their differential expression. From the miRMaster results page, the miEAA APIs for over-/underrepresentation and miRNA set enrichment analyses can be queried such that functional pathway enrichment analyses are feasible within minutes. Moreover, miRNAs are linked to target genes and target gene networks using miRTargetLink 2 (40), when applicable.

## RESULTS

In this section, we aim for a complete and self-containing description of miRMaster 2, partially sketching features that have already been available in the original version such as the miRNA prediction module. The improvements are highlighted, summarized in the conclusion and also marked in green in Figure 1.

### Data input

miRMaster relies on FASTQ files (optionally gzip compressed) as main input. Additionally, an annotation file can be provided by users to facilitate downstream analyses. While the annotation file is typically small, represented by few kilobytes of data, the FASTQ files can encompass several gigabytes per sample. Therefore, the data transfer for large studies could become a time-consuming task. We thus implemented an algorithm, exploiting the fact that miRNA-seq libraries are often of lower read complexity, that compresses the data typically by over 90%. This happens at the client side and only the compressed data are then transferred. Similarly, selected quality statistics are computed at the client side and only the relevant compressed information is transmitted. The server-side processing of the data starts immediately, i.e., while the data are transferred the processing is already initiated. We optimized these procedures in a way that studies with several hundred samples can be processed by miRMaster. The time-consuming analyses carried out at the server side afterwards are mostly implemented in efficient C++, such that even large-scale studies exceeding hundreds of samples are processed within a few hours.

### Supported organisms and RNA classes

While the original version of miRMaster was centered around the analysis of human microRNA data, supporting only a few other small RNA data analyses, we now provide full support for several other common organisms and more ncRNA classes. Importantly, the analysis of the RNA classes is not only restricted to small non-coding RNAs but also to longer RNAs such as circRNAs. With respect to the organisms, miRMaster 2 supports, besides *Homo sapiens*, *Mus musculus*, *Rattus norvegicus*, *Monodelphis domestica*, *Macaca mulatta*, *Gallus gallus*, *Bos taurus* and *Canis familiaris*.

### Pre-processing functionality and mapping

**Figure 2.**
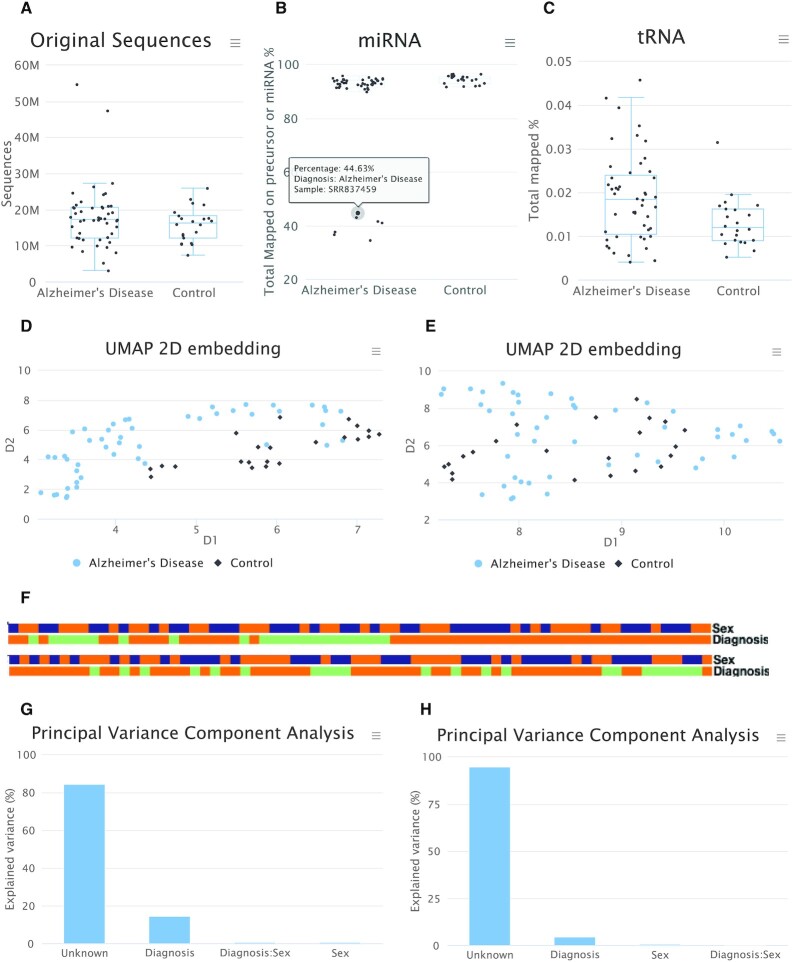
Selected result for the data pre-processing. The presented data are taken from the online demo data set on Alzheimer's Disease (AD). (**A**) Number of reads in the data set. Each dot represents a single sample. No difference between AD and controls exists. (**B**) Mapping to microRNAs. One point is highlighted. This feature can be used to identify outliers. (**C**) Mapping to tRNAs. In the overall distribution we observe here differences between the two classes. (**D**) Embedding of the samples using UMAP, colored by the disease phenotype using miRNAs. A clustering in the two groups can be recognized in this embedding. (**E**) The same embedding for tRNAs. In the case of this RNA class, no clear clustering is present. (**F**) Color-coded clustering for the sex and disease phenotype for microRNAs (top) and tRNAs (bottom). The two classes don’t show clustering with respect to sex but for microRNAs a clustering of AD samples can be observed. (**G**) Results of the PVCA for miRNAs. Around 15% of the total variance can be explained by the disease phenotype. (**H**) The same results for the tRNAs. Here, a lower percentage of variance is explained by the disease phenotype.

In the pre-processing step, adapters are trimmed and only sequences exceeding the selected minimum length are extracted. Furthermore the *GC* content is calculated. The results of this step are available as table and displayed as boxplots (Figure 2A). If the user provided several groups, as for instance cases and controls, or grades of severity for a disease, the information is displayed per group. In addition to the aggregated statistics, detailed per-sample statistics also are available. All tables can be downloaded in excel and csv format. All graphics are available as jpg, pdf, png and svg files. The underlying data for each graphic panel can also be downloaded in case users want to make figures on their own.

The second analysis step is genome and non-coding RNA mapping. First, the mapping to the reference genome is performed using the user specified input parameters and mapper. Mapping to the following 10 RNA classes is also performed: microRNA (Figure 2B), tRNA, piRNA, rRNA, scaRNA, lncRNA, snoRNA, snRNA, miscRNA and circRNA (Figure 2C). Additionally, mapping against viruses and bacteria from RefSeq is carried out for each sample, based on reads that did not map against the reference genome. As for the adapter trimming, all available information can be downloaded in excel and csv format and for each sample detailed mapping statistics are presented.

### Sample embedding, clustering, batch effect analysis

After completing the pre-processing and mapping, different aggregated analyses on the sample and RNA class level are carried out. First, an embedding using UMAP or alternatively PCA is available. The embedded graphics as 2D scatter plot can be colored with respect to arbitrary input variables extracted from the annotation file. For each RNA class, a distinct embedding is available (miRNA Figure 2D, tRNA Figure 2E). This allows users for example to visually compare whether for one RNA class a better clustering with respect to a disease phenotype is observed as compared to another.

Next, a sample correlation analysis is performed. Here, the correlation of the RNA expression between all pairs of samples is computed and shown as heatmap. The ordering of rows and columns can be modified and colored representations on top of the heatmaps show for each provided variable a color code (Figure 2F). Per default, hierarchical clustering with Euclidean distance and complete linkage is shown. In addition to the sample-to-sample correlation clustering, also the clustering of the expression values for each of the 10 RNA classes is computed, with the possibility to focus on the subset of RNAs with the largest variance. For both, rows (features) and columns (samples), clusters are defined and automatically adjusted. If a cluster is selected, the distribution of representatives within the cluster is displayed and *P*-values for enrichments are provided.

As last consideration of this functionality aspect, miRMaster 2 estimates the proportion of variance that can be attributed to variables provided in the annotation file. This can be biologically relevant metadata (again, case or control, or different severity grade of a disease, sex and many others) or it can be technical batches (e.g. the information which samples have been sequenced together or come from the same site in a multi-centric study). To this end, miRMaster 2 performs a PVCA, that combines aspects of principal component analysis as well as variance components analysis. The results are provided as bar charts, again for each of the RNA classes separately (Figure 2G for miRNA and Figure 2H for tRNAs). While miRMaster 2 highlights potential experimental batches it currently does not provide functionality to correct for batch effects. If experimental batches exist, a batch correction by the user is therefore recommended.

### Differential expression, downstream analysis and APIs to other tools

**Figure 3.**
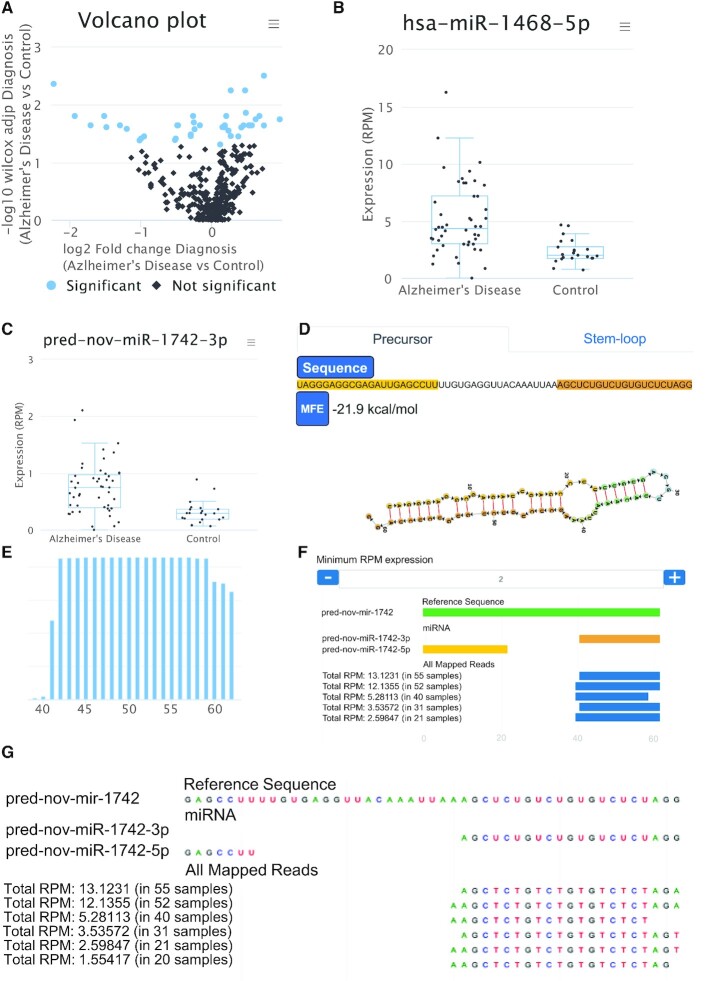
Downstream analyses for AD and control samples. (**A**) Volcano plot displaying each microRNA as a dot. Colored dots are statistically significant (adjusted *P*-value < 0.05). (**B**) For one significant marker (hsa-miR-1468–5p) the data are presented as boxplots. Again, single samples can be highlighted by moving the mouse over. (**C**) Box-plot for a novel miRNA candidate. (**D**) Precursor structure of this novel miRNA candidate and the minimum free energy. Users can switch between the representation of the precursor and the stem-loop. (**E**) Distribution of reads on the mature -3p miRNA of the same candidate precursor. Towards the end of the mature miRNA, the –3p heterogeneity that is typical for miRNAs can be recognized. (**F**) Representation of isoforms. The green bar denotes the precursor, the yellow bar the –5p mature form, the orange bar the –3p form. Each blue bar shows an isoform. The number of reads supporting the isoform can be dynamically adjusted by the user (here, at least 2 RPM are required). (**G**) If users zoom in the representation, the single base resolution per isoform is displayed (in this example, 1 RPM coverage is sufficient).

The previous analyses are based on sets of non-coding RNAs without focusing on single feature expression or differential expression, one of the key features of miRMaster 2. First, the expression of each miRNA is computed and shown in an aggregated and per-sample manner. The miRNAs can be sorted according to expression levels and pathway analyses using miEAA (all miRNAs) or miRTargetLink (single miRNA-gene interactions) are available. The results table can be downloaded as excel file or in csv format. If an annotation file was specified and a differential expression variable defined, volcano plots are generated. These show the negative decade logarithm of the *P*-value (adjusted Wilcoxon Mann–Whitney test) versus the log_2_ fold change (Figure 3A). From the interactive results table, miRNAs can be selected and boxplots detailing the expression of each sample are computed (Figure 3B/C). This table contains the raw and adjusted *P*-values of the Wilcoxon Mann–Whitney test as well as the results for a Shapiro Wilk normality test and *t*-test. If the data are normally distributed, t-test *P*-values can be used instead of the Wilcoxon Mann–Whitney test *P*-values. In addition, the table lists ANOVA and Kruskal–Wallis test *P*-values in case of more than two categories need to be compared per variable. As measure for the effect size, in addition to the fold change, the area under the receiver operator characteristics curve (AUC) is computed, as well as Cohen's d. For the de-re*g*ulated miRNAs, miRNA set enrichment analysis as well as over-representation analysis is facilitated through miEAA. The same metrics on differential expression are computed for each feature and all other RNA classes. In the case of other ncRNAs, however, no pathway enrichment analysis is available currently. This limitation might however be solved in the future by adding enrichment analyses for target genes or by integrating future data bases on pathways for other ncRNA classes that are developed.

A key question for researchers is to select the most valid de-regulated candidates and to exclude likely false positives. From our experience, several factors contribute to the success in validating de-regulated miRNAs (41). The miRNA has to show sufficient expression, relevant effect sizes between cases and control and at best a statistically significant difference. To allow users filtering for the best candidates, the results table offers the option to add several filter criteria that are connected by a logical ‘and’. Authors can filter for those miRNAs present with at least 1 RPM, having an effect size of at least 0.7 and a *P*-value <0.05. By adapting the parameters, users can balance for rather specific results or rather sensitive results, depending on the underlying biological question.

Remarkably, miRMaster 2 supports the analysis of multiple comparisons at the same time. If the annotation column that is selected by the user has for example four groups, all pair-wise comparisons (4 × 3/2 = 6) are carried out and presented to the user. Currently, however, only one annotation column can be used at a time for an analysis to avoid too many computations and complicated result representations.

The last analysis module comprises the prediction of new miRNAs. Again, the same information on expression and de-regulation as for the 10 RNA classes is calculated and presented to the user. Here, novel precursor miRNAs found in the data can be selected. An example is a precursor miRNA where the 5′ mature form is annotated while the 3′ that is expressed in the user's data is not yet annotated. For each miRNA candidate, the secondary structure and free energy is computed and presented (Figure 3D). Finally, the expression of reads on the 5′ and 3′ mature form (Figure 3E) is provided as bar-plot along the precursor. Here, users can verify the read stacks manually and in principle observe potential 3′ heterogeneity. Next, all the mapping reads are presented, containing potential isomiRs or other RNA fragments (Figure 3F). This module offers full-download capabilities as enumerated for the other analysis modules.

We clearly consider the respective potentially new miRNAs as candidates only. It is frequently hard to distinguish between molecules of the different ncRNA classes (e.g. miRNAs that are actually tRNA fragments) or to exclude artifacts. The candidates call for an in-depth experimental validation before they should be considered as miRNAs (42). The validation rate is tightly coupled with the quality of the pre-selected precursors. In our previous study we reached a validation of almost 20%.

In the following sections we provide three use cases, first the analysis of a sncRNA-seq data set from *Mus musculus*, second, human sncRNA data from dementia patients and finally the analysis of single-cell small non-coding RNA data using unique molecular identifiers. All use cases are available on the miRMaster homepage.

### Use Case (1): *Mus musculus* sncRNA atlas

**Figure 4.**
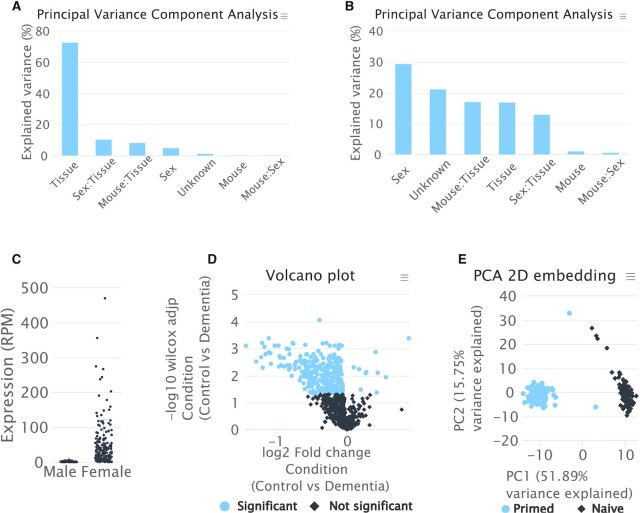
Downstream analysis results for the use cases. (**A**) Principal Variance Component Analysis based on the miRNA expression matrix, showing most of the variance explained by the mouse tissue. (**B**) Principal Variance Component Analysis based on the circRNA expression matrix, showing most of the variance explained by the mouse sex. (**C**) Reads per million (RPM) normalized expression of mmu_circ_0004351. (**D**) Volcano plot showing significantly deregulated miRNAs in dementia patients. (**E**) PCA embedding of the miRNA expression matrix showing a perfect separation between primed and naïve hESCs.

To demonstrate the analysis scope of miRMaster for a non-human species, we analyzed sncRNA sequencing data from *M. musculus* (43). Here, for 11 organs and up to 14 replicates of both sexes, sncRNA data using Illumina sequencing have been analyzed. We started miRMaster with the 272 FASTQ files downloaded from SRA, used the tissue and mouse ID as annotation and configured the sex as differential expression variable. The complete results were available after 4 hours. The pre-processing page highlights a large span of sequencing depth, going from a few thousands up to 25 million reads, with a median of 1.9 million reads for the male samples and 5.1 million reads for the female samples. The mapping statistics show overall similar patterns between male and female samples for all considered RNA classes. Interestingly, the miRNA mapping statistics show the highest variability, going from less than 5% mapped reads for some samples, up to over 90% of mapped reads. This variability can mainly be attributed to the RNA composition of the different tissues, as shown by the batch analysis, where 73% of the observed variance can be explained by the tissue variable (Figure 4A). We find that for all other RNA classes, although varying in the proportion of explained variance, the tissue is the strongest factor, except for circRNAs, where most of the observed variance is explained by the sex (Figure 4B). These results are reflected by the PCA and UMAP embeddings, as well as the sample correlation and expression clustering. The circRNA showing the most significant change between male and female mice was mmu_circ_0004351, with a fold change of 17.5 and an FDR adjusted Wilcoxon Mann-Whitney *P*-value of 4.6 × 10^–32^ (Figure 4C). miRNAs for which only 5% of the observed variance could be attributed to the mouse sex, were also partially differentially expressed, and have been previously reported in literature for single tissues or other species, such as mmu-miR-27a-3p and miR-27b-3p (44,45) and miR-16-5p and miR-21-3p (46).

### Use Case (2): Differential sncRNA expression in neurodegeneration

We previously analyzed the role of microRNAs in Alzheimer's disease (4,47,48). Moreover, the examples shown above are from Alzheimer's patients and controls (Figures 2 and 3). Recently, we published a data set composed of dementia patients, including Alzheimer's disease and controls with a new technology, termed CoolMPS (49). To demonstrate the functionality of miRMaster for this sequencing assay, we analyzed the 216 case and control samples. The pre-processing page shows that all samples have been sequenced with more than 13 million reads, obtaining a median of 27.5 million for all samples. The genome mapping statistics show high mapping rates with a median of 95.5% and only few samples exhibiting a mapping rate below 90%. Since the RNAs of this data set were size selected to enrich for miRNAs, we observe the expected high mapping rates to miRNAs as well, with a median of 90.0%. The batch effect analysis suggests that most of the variation cannot be explained by any of the provided variables, followed by the age group and condition for most RNA classes. This is reflected by the sample embeddings as well as sample and expression clusterings, since no separation according to any of the provided annotations can be achieved. The differential expression results of the RNA classes show a general trend of over-expression in dementia patients, since most RNAs are down-regulated in control patients. We find that 695 miRNAs were expressed with at least 3 reads in more than 50% of either control or dementia patients and that 270 miRNAs were significantly de-regulated with an adjusted Wilcoxon Mann-Whitney *P*-value below 0.05 (Figure 4D). Upon triggering an enrichment analysis with miEAA 2.0, we find 1,790 affected categories comprising known dementia and Alzheimer's disease related pathways such as the positive regulation of endoplasmic reticulum unfolded protein response (FDR adjusted *P*-value of 0.003, (50)) and Rab GTPase binding (FDR adjusted *P*-value 0.001, (51)).

### Use Case (3): analysis of data with unique molecular identifiers

Due to the increasing popularity of single cell small non-coding RNA sequencing, we also demonstrate the capabilities of miRMaster on 168 primed and naïve hESCs sequenced with a UMI-based protocol presented by Faridani *et al.* (52). Based on the pre-processing of miRMaster we find that the sequencing depth distribution is similar between primed and naïve hESCs and ranges from 1 million reads up to 44 million with a median of 4.2 million. As expected for small input protocols, and especially in the context of single cell miRNA-seq, only about 50% of the reads were kept after adapter trimming and quality filtering, which can often be attributed to adapter dimerization. The genome mapping statistics show a large variability going from 4.4% up to 88.3%, where on median 0.9% of the reads mapped against microRNAs. The most represented RNA class are rRNAs (median 6.3%) followed by snoRNAs with a median of 3.8%. The batch effect analysis shows that most of the variance in miRNA and snoRNA counts is associated with the cell state (76.3% and 82.9%), in contrast to the other RNA classes, where most of the variance cannot be explained by the cell state. This separation is clearly displayed by the miRNA PCA embedding, where the first component almost perfectly splits the primed from the naïve cells (Figure 4E). The miRNA sample correlation matrix shows high similarity (>0.7) between the cells of each state, while the correlation between the groups is in the range between 0.2 and –0.2. The correlation clustering shows similar patterns for the snoRNAs, however, the correlation coefficients inside the same cell state are higher and in-between cell states vary in the range of 0.7 and 0.85. As highlighted in the publication by Faridani *et al.*, the most up-regulated miRNA in naïve hESCs is hsa-miR-371a-5p (fold change of 797, FDR adjusted *P*-value of 1.11×10^–27^), whereas the most down-regulated miRNA is hsa-miR-363–3p (fold change of 0.003, FDR adjusted *P*-value of 1.07 × 10^–29^). It is evident that the current comparably shallow single cell small RNA data sets are not sufficient to detect all the non-coding RNA molecules present in a single cell. Especially the lower abundant non-coding RNA classes might not be sufficiently represented given the current experimental limitations. Nonetheless, miRMaster 2 identifies the molecules that are present in the sequencing data, representing a functional single cell non-coding RNA analysis pipeline.

## CONCLUSION AND FUTURE DIRECTION

One of the most important novel features is certainly the support for multiple species, with which we expect to largen our userbase. Furthermore, we added circRNAs as new RNA class. Moreover, the scope of the analysis modules has been widened and we offer new data analysis aspects, such as (i) sample correlation, (ii) expression clustering, (iii) embedding, (iv) batch effect assessment and (v) differential expression analyses. Finally, we extended the adapter trimming procedure to support more major sncRNA-seq library protocols and other custom protocols. In the same context, we implemented support for UMI based analysis, thereby making the tool ready for single cell sncRNA data. In addition to the new features, we performed a major update of the underlying data bases to their current standards, improved the user-experience as well as the representation of results in tables and as interactive plots.

While we incorporated a lot of feedback from researchers that used miRMaster in this update and therefore implemented new features that were missing also from our own experience, we still see potential to further develop the usability and scope of miRMaster. One aspect that we plan to improve is automated quality control. This includes a prediction of the uploaded sample type. By using annotated data, the solid tissue or body fluid from which the data were generated can be predicted with over 90% accuracy (43). A further step is to improve the functional support for viruses and bacteria. Originally, we implemented this step for detecting contamination or reporting the presence of exogenous species. While we tested this feature only using expression data from Myxobacteria, we now aim to design a dedicated analysis module for sRNAs from microorganisms. In our ambition to develop an AI-based quality control we plan to implement automated outlier detection and propose users to perform the computational analyses after excluding flagged outliers.

To further advance the development of miRMaster, we encourage the community to continue providing us constant feedback as well as to propose new features that are of broad interest.

## DATA AVAILABILITY

miRMaster 2 is freely available at https://www.ccb.uni-saarland.de/mirmaster2.
